# Neurogenin 3 is regulated by neurotrophic tyrosine kinase receptor type 2 (TRKB) signaling in the adult human exocrine pancreas

**DOI:** 10.1186/s12964-016-0146-x

**Published:** 2016-09-22

**Authors:** Michael J. Shamblott, Marci L. O’Driscoll, Danielle L. Gomez, Dustin L. McGuire

**Affiliations:** 1 Department of Pediatrics, Children’s Research Institute, University of South Florida Morsani College of Medicine, 601 4th St. South, CRI 3005, St. Petersburg, FL 33701 USA; 2 Morphogenesis, Inc, 4613 N. Clark Ave, Tampa, FL 33614 USA

**Keywords:** TRKB, NTRK2, Neurogenin 3, NGN3, Pancreas, Exocrine, Endocrine, AKT, Protein kinase B

## Abstract

**Background:**

Reports of exocrine-to-endocrine reprogramming through expression or stabilization of the transcription factor neurogenin 3 (NGN3) have generated renewed interest in harnessing pancreatic plasticity for therapeutic applications. NGN3 is expressed by a population of endocrine progenitor cells that give rise exclusively to hormone-secreting cells within pancreatic islets and is necessary and sufficient for endocrine differentiation during development. In the adult human pancreas, NGN3 is expressed by dedifferentiating exocrine cells with a phenotype resembling endocrine progenitor cells and the capacity for endocrine differentiation in vitro. Neurotrophic tyrosine kinase receptor type 2 (TRKB), which regulates neuronal cell survival, differentiation and plasticity, was identified as highly overexpressed in the NGN3 positive cell transcriptome compared to NGN3 negative exocrine cells. This study was designed to determine if NGN3 is regulated by TRKB signaling in the adult human exocrine pancreas.

**Methods:**

Transcriptome analysis, quantitative reverse transcriptase polymerase chain reaction (RTPCR) and immunochemistry were used to identify TRKB isoform expression in primary cultures of human islet-depleted exocrine tissue and human cadaveric pancreas biopsies. The effects of pharmacological modulation of TRKB signaling on the expression of NGN3 were assessed by Student’s *t*-test and ANOVA.

**Results:**

Approximately 30 % of cultured exocrine cells and 95 % of NGN3+ cells express TRKB on their cell surface. Transcriptome-based exon splicing analyses, isoform-specific quantitative RTPCR and immunochemical staining demonstrate that TRKB-T1, which lacks a tyrosine kinase domain, is the predominant isoform expressed in cultured exocrine tissue and is expressed in histologically normal cadaveric pancreas biopsies. Pharmacological inhibition of TRKB significantly decreased the percentage of NGN3+ cells, while a TRKB agonist significantly increased this percentage. Inhibition of protein kinase B (AKT) blocked the effect of the TRKB agonist, while inhibition of tyrosine kinase had no effect. Modulation of TRKB and AKT signaling did not significantly affect the level of NGN3 mRNA.

**Conclusions:**

In the adult human exocrine pancreas, TRKB-T1 positively regulates NGN3 independent of effects on NGN3 transcription. Targeting mechanisms controlling the NGN3+ cell population size and endocrine cell fate commitment represent a potential new approach to understand pancreas pathobiology and means whereby cell populations could be expanded for therapeutic purposes.

**Electronic supplementary material:**

The online version of this article (doi:10.1186/s12964-016-0146-x) contains supplementary material, which is available to authorized users.

## Plain English summary

Neurogenin 3 (NGN3) is a transcription factor that is essential for development of endocrine hormone-producing pancreatic islet cells. NGN3 also is expressed by dedifferentiated cells in the adult human exocrine pancreas that have begun to take on characteristics of endocrine progenitor cells during development. Neurotrophic tyrosine kinase receptor type 2 (TRKB), a cytokine receptor that promotes functional plasticity in cells of the developing and adult nervous system, was identified as being overexpressed by the NGN3+ cell population compared to NGN3- exocrine cells. In this report we demonstrate that NGN3+ cells in the adult human exocrine pancreas express TRKB-T1, an isoform that lacks a tyrosine kinase domain. Positive regulation of NGN3 protein by TRKB involves the cell-signaling molecule protein kinase B (AKT). Blocking tyrosine kinase activity had no effect on the percentage of cells expressing NGN3 protein. Pharmacological methods that target the NGN3+ cell population size and endocrine cell fate commitment may be an approach to treat diabetes.

## Background

The transcription factor neurogenin 3 (NGN3) is necessary and sufficient for islet specification during development [1–4]. NGN3 also is expressed by acinar and duct cells in histologically normal pancreas biopsies and in primary cultures of adult human exocrine tissue [5], but has not been detected in the adult rodent pancreas. NGN3+ cells isolated by coexpression of the cell surface glycoprotein CD133 from adult human exocrine tissue have a transcriptome consistent with exocrine-cell dedifferentiation, a phenotype that resembles endocrine progenitor cells during development and a capacity for endocrine differentiation in vitro [5]. Comparison of the NGN3+/CD133+ cell transcriptome to surrounding NGN3/CD133-depleted (NGN3D/CD133D) exocrine cells identified significant upregulation of neurotrophic tyrosine kinase receptor type 2 (NTRK2, referred to as TRKB) [5]. TRKB regulates neuronal cell function, differentiation, survival and plasticity [6–8] and is expressed in the pancreas [9, 10] and by pancreatic ductal adenocarcinoma cells [11–15]. The human TRKB gene encodes at least 100 unique transcripts and 10 protein isoforms with distinctive functional domains and downstream signaling pathways generated through alternate promoters, gene splicing and stop codon usage [16]. The full-length isoform (TRKB-FL) signals through a tyrosine kinase domain that leads to downstream activation of protein kinase B (AKT) [17, 18], extracellular signal related kinase (ERK) [19, 20] and phospholipase C [21, 22]. The TRKB-T1 isoform, which lacks a tyrosine kinase domain [23–26], can form heterodimers with TRKB-FL to reduce tyrosine kinase activity [27] or positively signal through the p75 low-affinity nerve growth factor receptor [28], AKT [29] or Rho guanine dissociation inhibitor and Rho/Rac GTPases [30, 31].

Here, we demonstrate that TRKB-T1 is the predominant isoform expressed in cultured adult human exocrine tissue and is coexpressed with NGN3 in cadaveric pancreas biopsies. In primary cultures of human exocrine tissue, the percentage of NGN3+ cells is positively regulated by TRKB signaling. Inhibition of AKT, but not tyrosine kinase, blocks the regulation of NGN3 by TRKB. TRKB-T1 signaling in NGN3+ cells may contribute to cellular dedifferentiation indicative of exocrine cell plasticity. As such, the mechanisms regulating NGN3 may provide insights into pancreas pathobiology and the therapeutic potential of this cell population.

## Methods

### Primary exocrine tissue culture

Islet-depleted cadaveric exocrine tissue and histologically normal biopsies from human pancreata of non-diabetic adult cadaver donors was obtained from ICR Basic Science Islet Distribution Program (IIDP) under the auspices of the National Institutes of Health National Center for Research Resources (NCRR). Written informed consent for research use was obtained by the institutions that collected the tissue. Tissues were received without personal identifiers. Exocrine tissue was received within 48 h post mortem and cultured at a density of 100 ml media per ml of pelleted tissue in serum-free Miami Media 1A (Mediatech 98-021-CV, Manassas, VA, USA) supplemented with freshly prepared 0.01 g L^-1^ reduced glutathione. Tissue was plated in low-adhesion plastic dishes and maintained in suspension culture at 37 °C, 5 % CO_2_ for 4–6 days with media replacement every 2 days. Drugs were administered initially, at media replenishment and 1–2 h before harvest. Drugs and final dosages were: 7,8-dihydroxyflavone (0.1 μM, Tocris Bioscience, Bristol, UK); N-[2-[[(Hexahydro-2-oxo-1H-azepin-3-yl)amino]carbonyl]phenyl]benzo[b]thiophene-2-carboxamide (ANA-12, 50 μM, Tocris Bioscience, Bristol, UK); 4-Amino-5,8-dihydro-5-oxo-8-β-D-ribofuranosyl-pyrido[2,3-d]pyrimidine-6-carboxamide (API-1, 1 μM, Tocris Bioscience, Bristol, UK); (9S, 10S, 12R)-2,3,9,10,11,12-Hexahydro-10-hydroxy-10-(hydroxymethyl)-9-methyl-9,12-epoxy-1H-diindolo[1,2,3-fg:3’,2’,1’-kl]pyrrolo[3,4-i] [1, 6]benzodiazocin-1-one (CEP-701, 10 nM, Sigma-Aldrich, St. Louis, MO, USA) and 5-(2-Phenyl-pyrazolo[1,5-a]pyridin-3-yl)-1H-pyrazolo[3,4-c]pyridazin-3-ylamine (FR180204, 20 μM, Tocris Bioscience, Bristol, UK). All drugs were resuspended in dimethyl sulfoxide (DMSO). Drug dosages were the maximum possible without causing substantial cell death as determined by ethidium bromide exclusion and esterase activity (LIVE/DEAD, Life Technologies, Carlsbad, CA, USA) and nuclear morphology. Replication (n) refers to biological replicate exocrine cultures.

### FACS analyses

To measure expression of TRKB, live single cell suspensions of cultured exocrine tissue were stained with mouse anti-human TRKB (R&D Systems, Minneapolis, MN, USA 1:20) or mouse IgG1 isotype negative control (BD Pharmingen, San Jose, CA, USA #340761 1:10) in phosphate-buffered saline (PBS), pH 7.2, 0.5 % bovine serum albumin (BSA, Jackson ImmunoResearch, West Grove, PA, USA), and 2 mM ethylenediaminetetraacetic acid (EDTA) for 30 min at 4 °C. Cells were then washed and stained with anti-mouse Alexa Fluor-647 (Invitrogen, Carlsbad, CA, USA) for 30 min at 4 °C.

To measure coexpression of TRKB and NGN3, cells were then fixed, blocked and permeabilized using Transcription Factor Buffer Set reagents (BD Pharmigen, San Jose, CA, USA) and stained with rabbit anti-human NGN3 (Sigma, St. Lois, MO, USA Prestige HPA039785, 1:500), or an equivalent concentration of rabbit Ig negative control, overnight at 4 °C. NGN3 staining was detected with anti-rabbit Alexa Fluor-488 (Invitrogen, Carlsbad, CA, USA) secondary antibody for 30 min at 4 °C. Cell populations were gated on FL1/SSC-A to identify the NGN3+ cell population and on FL4/SSC-A to identify the TRKB+ population. To quantify percentage of NGN3+ cells that are TRKB+, NGN3 gated cells were analyzed for TRKB expression in FL4. To quantify the percentage of TRKB+ cells that are NGN3+, TRKB gated cells were analyzed for NGN3 expression in FL1.

To measure coexpression of TRKB and CD133, cells were then stained with mouse anti-CD133-phycoerythrin (PE) (Miltenyi Biotec, San Diego, CA, USA 1:10) or mouse IgG2b-PE isotype negative control (BD Pharmingen, San Jose, CA, USA #555743 1:10) for 10 min at 4 °C. Staining for TRKB and anti-mouse secondary antibody prior to staining for CD133 with an antibody conjugated to PE was used to avoid cross-reactivity between the two mouse monoclonal primary antibodies. Cell populations were gated on FL2/SSC-A to identify the CD133+ population and on FL4/SSC-A to identify the TRKB+ population. To quantify the percentage of CD133+ cells that are TRKB+, CD133 gated cells were analyzed for TRKB expression in FL4. To quantify the percentage of TRKB+ cells that are CD133+, TRKB gated cells were analyzed for CD133 expression in FL2. Gating results shown in Additional file 1: Figures S1 and Additional file 2: Figure S2.

### Tissue immunohistochemistry

Cadaveric pancreas wedge biopsies frozen at -80 °C 1–5 h post mortem and cultured exocrine tissue were embedded in OCT mounting media (Tissue-Tek, Sakura, Torrence, CA, USA) then snap frozen in a dry ice/2-methylbutane bath. Tissues were stored at -80 °C and sectioned as soon as possible after embedding. Eight micron frozen sections were fixed for 5 min in 4 % paraformaldehyde in PBS, quenched for 5 min in 50 mM glycine in PBS then blocked in 5 % donkey serum (Jackson ImmunoResearch, West Grove, PA, USA), 1 % BSA, 0.1 % Triton-X100 in PBS for 30 min at room temperature. Detection of NGN3 was carried out with a mouse monoclonal antibody (Developmental Studies Hybridoma Bank, Iowa city, IA, USA F25A1B3 hybridoma supernatant, 1:10) or rabbit polyclonal antibody (Sigma St. Lois, MO, USA Prestige HPA039785, 1:500). TRKB-T1 isoform was detected using a rabbit polyclonal antibody (Santa Cruz, Santa Cruz, CA, USA Sc-119, 1:250). Secondary antibodies were donkey antisera to the primary antibody conjugated to Alexa Fluor-488 or -546 (Invitrogen, Carlsbad, CA, USA) and counterstained with Hoechst 33342 (Invitrogen, Carlsbad, CA, USA) to visualize nuclei.

Quantitative immunohistochemistry was carried out by imaging 10 random fields (technical replicates) of >200 nuclei per field spanning >100 microns of tissue depth for each treatment group. Fields were captured with Metamorph morphometry software (Metamorph, Sunnyvale, CA, USA) and nuclei were counted using ImageJ image analysis software (http://imagej.nih.gov) blinded to treatment. Some drug treatment studies were carried out independently but are shown together. Significance was determined using a two-tailed homoscedastic Student’s *t*-test between drug and carrier control for single drug studies and using a one-way ANOVA with Bonferroni post hoc testing for multiple drug studies. Sample size (n) refers to biological replicate exocrine cultures. Results were reported as mean ± SEM (standard error of the mean) of technical and biological replicates.

### Western blot analyses

Western blots were incubated in Li-cor blocking solution (Li-cor Bioscience, Lincoln, NE, USA) 1 h at room temperature then stained with primary antibodies in 5 % BSA, PBS, 0.1 % Triton-X100 overnight at 4 °C. Antibodies and dilutions were: total AKT (Cell Signaling, Beverly, MA, USA #9272, 1:1000), p(S473)AKT (Cell Signaling, Beverly, MA, USA #4060, 1:1000), total ERK1/2 (Cell Signaling, Beverly, MA, USA #4695, 1:1000), p(T202/Y204) ERK1/2 (Cell Signaling, Beverly, MA, USA #4370, 1:1000), GAPDH (Millipore, Billerica, MA, USA MAB374, 1:5000), pan-TRKB (R&D Systems, Minneapolis, MN, USA MAB3971, 1:250), TRKB-T1 (Santa Cruz, Santa Cruz, CA, USA SC-119, 1:1000). Bound proteins were detected with goat anti-mouse or goat anti-rabbit secondary antibodies conjugated to Dylite-680 or Dylite-800 (Thermo Fisher Scientific, Waltham, MA, USA) diluted 1:10,000 in Li-cor blocking buffer (Li-cor Bioscience, Lincoln, NE, USA) with 0.1 % Tween-20, 0.05 % sodium dodecyl sulfate at 25 °C for 1 h then imaged on an Odyssey infrared imager (Li-cor Bioscience, Lincoln, NE, USA). For quantification of western blot results, a Student’s *t*-test (*n* = 3) was performed on band intensities following staining with anti-p(S473)AKT divided by band intensities following staining with total AKT.

### Quantitative RTPCR

RNA from cultured exocrine tissue and isolated cells were prepared using the RNeasy miniprep kit (Qiagen, Venlo, Netherlands). cDNA was synthesized using oligo(dT) primers in a standard reverse transcriptase reaction with 5 μg RNA. mRNA levels were determined by using TaqMan gene expression assays (Applied Biosystems, Foster City, CA, USA): NGN3 (Hs01875204_s1), Pan-TRKB (Hs00178811_m1), TRKB-FL (Hs01093096_m1), TRKB-T1 (Hs01093110_m1), HES1 (Hs00172878_m1), KI-67 (Hs01032443_m1) and primer-attenuated cyclophillin A (PPIA, 4310883E). Expression was calculated as mean ± SEM 2^-ΔΔC^_t_ of 5 technical replica readings for each biological replicate exocrine culture. Each technical replica reaction had approximately 50 ng cDNA template. TRKB expression was normalized to PPIA and expressed as percent of total TRKB level, which was determined separately for exocrine tissue and isolated cells. A mean threshold cycle (C_t_) of >35 was treated as no expression.

### Transcriptome analyses

Random-primed cDNA from CD133+ and CD133-depleted populations (*n* = 3 biological replicate exocrine cultures) were subjected to >20 million DNA sequencing reads per sample on an HiSeq2000 (Illumina, San Diego, CA, USA) sequencing system then analyzed using the TopHat-Cufflinks-Cuffdiff workflow [32]. Sequencing data was mapped with TopHat (v2.0.5) against the UCSC hg19 reference assembly and viewed using the Integrated Genome Viewer (IGV, broadinstitute.org). Sashimi plot analysis was carried out using IGV to quantify TRKB exon splicing from the transcriptome dataset. Transcriptome data is accessible at the NCBI Gene Expression Omnibus through accession number GSE64854. Neurotrophin pathway activity analysis was done using CummeRbund analysis software [33]. The neurotrophin pathways list is comprised of 320 genes directly involved in neurotrophin signaling or downstream targets curated from the PathCards database (Weizmann Institute of Science, pathcards.genecards.org), SA Biosciences RT2 array list (sabiosciences.com) and the Broad Institute Molecular Signatures Database (MSigDB, broadinstitute.org). Neurotrophin pathway activity score was calculated as the percentage of genes that were upregulated or down regulated by more than 2-fold / the number of genes on the list of 320 that were expressed. Pathway score significance (p) was determined by a one sample two-tailed Student’s *t*-test against 10 scores from randomly selected and equally sized gene lists matched to the expression dataset.

## Results

### NGN3+ exocrine cells coexpress TRKB and CD133

**Fig. 1:**
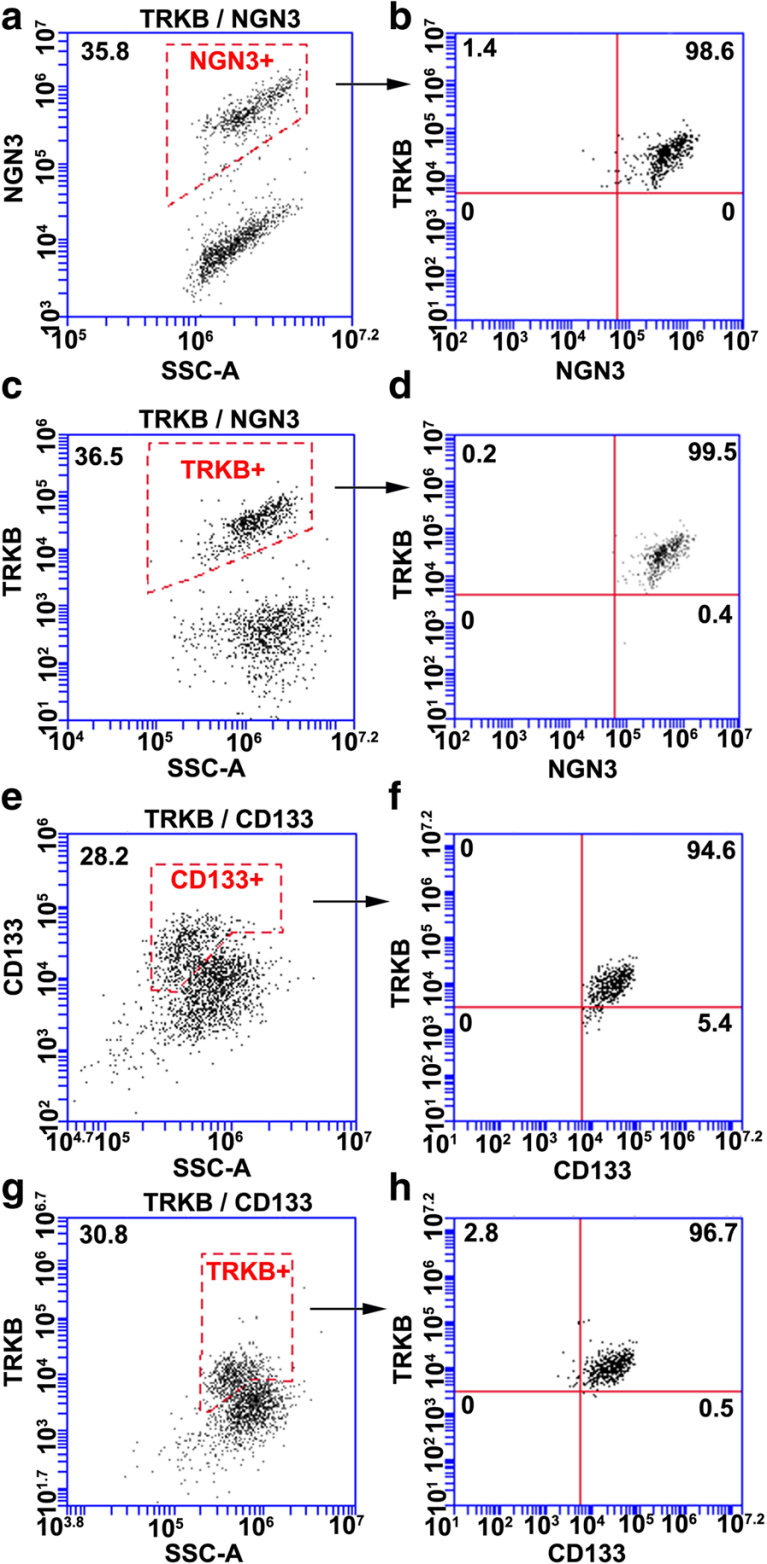
FACS analysis of neurotrophic tyrosine kinase receptor type 2 (TRKB) expression in cultured human exocrine tissue. Single cells from a primary suspension culture of adult human exocrine tissue were stained with anti-TRKB and anti-mouse Alexa Fluor-647 then fixed, permeabilized and stained with anti-NGN3 (**a**-**d**). **a** NGN3+ population gated based on isotype negative control. **b** TRKB/NGN3 coexpression of population gated in **a**. **c** TRKB+ population gated based on isotype negative control. **d** TRKB/NGN3 coexpression of population gated in **c**. Single cells from a biological replicate exocrine tissue culture were stained with anti-TRKB and anti-mouse Alexa Fluor-647 then stained with anti-CD133-PE (phycoerythrin) (**e**-**h**). **e** CD133+ population gated based on isotype negative control. **f** TRKB/CD133 coexpression of population gated in **e**. **g** TRKB+ population gated based on isotype negative control. **h** TRKB/CD133 coexpression of population gated in **g**. Antibody combination shown above plots. Gates shown with dashed and solid red lines. Percentage of cells in each gate are shown in upper corner

TRKB and NGN3 were expressed by a mean ± SEM of 28.2 ± 4.3 % and 28.2 ± 4.4 % of cells, respectively, in three biological replicate primary cultures of adult human exocrine tissue. In a representative culture, 35.8 % and 36.5 % of cells were positive for NGN3 and TRKB, respectively and approximately 99 % of cells coexpressed both proteins (Fig. 1a–d). NGN3+ cells can be isolated from human exocrine tissue by immunomagnetic selection for coexpressed cell surface marker CD133 [5]. In a representative culture, 28.2 % and 30.8 % of cells were positive for CD133+ and TRKB+, respectively and there was approximately 95 % coexpression of both proteins (Fig. 1e–h) (FACS controls and gating strategies in Additional file 1: Figure S1 and Additional file 2: Figure S2). Coexpression results were replicated in two additional exocrine cultures. Taken together with an approximately 270-fold enrichment of TRKB mRNA in isolated NGN3+/CD133+ cells compared to the NGN3D/CD133D population [5], these FACS results demonstrate extensive expression of TRKB by NGN3+/CD133+ cells in cultured adult human exocrine tissue.

### NGN3+ exocrine cells express the TRKB-T1 isoform

**Fig. 2:**
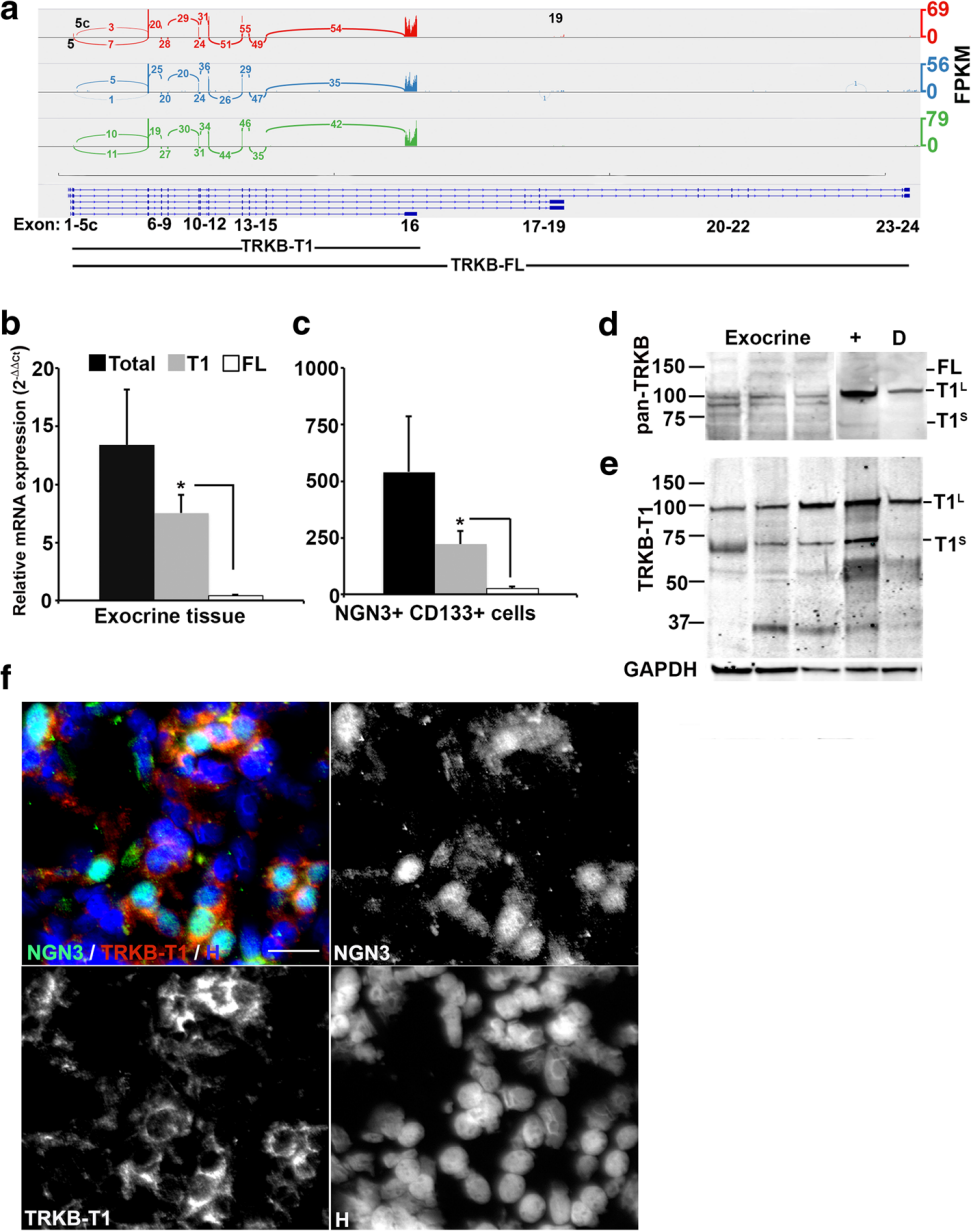
Quantitative analyses of TRKB isoform expression in cultured human exocrine tissue. **a** Isoform splicing of the neurotrophic tyrosine kinase receptor type 2 (TRKB) gene by NGN3+ cell populations isolated by coexpression of CD133 from 3 biological replicate exocrine cultures. TRKB loci and major isoforms shown below. Number of reads crossing exons are shown along with lines indicating joined exons. Height of peak indicates relative expression level in fragments per kilobase of transcript per million mapped reads (FPKM). Usage of alternate transcriptional start sites in exons 5 and 5c and alternate polyadenylation and stop codon in exon 19 are highlighted. **b**, **c** mRNA levels of total TRKB, TRKB-T1 and TRKB-FL isoforms. Relative Isoform expression calculated as mean ± SEM (*n* = 3 biological replicate cultures) of 2^-ΔΔC^_t_ normalized to the level of cyclophillin A (PPIA) in (**b**) exocrine tissue and (**c**) NGN3+/CD133+ cells isolated from exocrine tissue. *, *p* < 0.05. TRKB levels in NGN3/CD133-depleted exocrine cells were too low to detect. **d**, **e** Western blot analyses of TRKB protein isoform expression. Protein lysates from 3 biological replicate exocrine cultures and NGN3+/CD133+ (+) and NGN3/CD133-depleted (*D*) cells from a single exocrine culture were probed with: **d** pan TRKB-specific TRKB antibody and (**e**) TRKB-T1-specific antibody. Apparent molecular mass for TRKB-FL, TRKB-T1 large size variant (T1^L^) and TRKB-T1 small size variant (T1^S^) shown at right. Molecular weight markers shown on left in kDa. Level of glyceraldehyde phosphate dehydrogenase (GAPDH) used as loading control. **f** Representative immunohistochemical staining of human cadaveric pancreas biopsy tissue for expression of NGN3 and TRKB-T1. Color overlay and individual monochrome images are shown with antibodies used. Nuclei counterstained with Hoechst 33342 (*H*). Scale bars are 20 microns

Transcriptome analyses and quantitative RTPCR were used to identify TRKB isoform expression in cultured exocrine tissue. Abundant transcription, as indicated by peak height and number of reads joining each exon, of TRKB-T1-specific exon 16 and low level expression of TRKB-FL exons 17–24 in each of three biological replicate NGN3+/CD133+ cell transcriptomes demonstrate that TRKB-T1 is the predominant mRNA transcript expressed in this population. Transcription of alternate exons 5c and 19 indicate low levels of TRKB-N-T1 [23] and TRKB-T-Shc isoforms, respectively (Fig. 2a).

Isoform-specific quantitative RTPCR was used to quantify the relative transcription levels of total TRKB, TRKB-FL and TRKB-T1. TRKB-T1 expression was significantly higher than TRKB-FL in three biological replicate exocrine cultures (*p* = 0.01) (Fig. 2b) and in three biological replicate paired NGN3+/CD133+ and NGN3D/CD133D cell populations (*p* = 0.03) (Fig. 2c) in agreement with transcriptome analyses. The expression level of TRKB by NGN3D/CD133D cells was too low to be detected by transcriptome or quantitative RTPCR analyses (mean C_t_ >35 cycles).

Multiple TRKB isoforms also were observed in western blot analyses of protein lysates from three biological replicate exocrine cultures as well as lysates from paired NGN3+/CD133+ and NGN3D/CD133D cell populations. Immunostaining with a pan TRKB-specific antibody detected a weak band at approximately 140 kDa, which corresponds to the predicted size of TRKB-FL [34], in exocrine tissue but not isolated cell extracts (labeled FL in Fig. 2d). Human TRKB-T1 has been detected at either 90–95 kDa [35] or 60–70 kDa [12, 23]. A band corresponding to the larger TRKB-T1 variant or to TRKB-T-Shc [23] (labeled T1^L^ in Fig. 2d,e) can be detected in exocrine tissue, NGN3+/CD133+ and to a lesser extent NGN3D/CD133D cell lysates, while the smaller variant (labeled T1^S^ in Fig. 2d,e) was weakly detected in NGN3+/CD133+, but not NGN3D/CD133D cell extract (Fig. 2d). Western blot analysis using a TRKB-T1-specific antibody identified a band migrating at approximately 37 kDa, which corresponds to the predicted molecular weight of TRKB-N-T1 (Fig. 2e), and confirmed the presence of both TRKB-T1 size variants in protein lysates from three biological replicate exocrine tissue cultures and a single paired set of NGN3+/CD133+ cell extracts. Western blot results using pan TRKB and TRKB-T1 specific antibodies were replicated in a second paired set of isolated cell extracts. Expression of TRKB-T1 by NGN3+ cells was also detected in four biological replicate human cadaveric pancreas biopsies (representative images shown in Fig. 2f).

Whereas the TRKB-T1^S^ size variant was enriched in the NGN3+/CD133+ cell population, detection of TRKB-T1^L^ in both NGN3+/CD133+ and NGN3D/CD133D cell extracts conflicts with low or undetectable levels of TRKB detected in the NGN3D/CD133D population by FACS, transcriptome, and quantitative RTPCR. As it is detected in NGN3D/CD133D extract (Fig. 2d sample D), the TRKB-T1^L^ band may reflect non-specific binding or represent a form of TRKB not readily detected in the transcriptome or by PCR and antibody reagents. Irrespective of the identity of this band, these results demonstrate TRKB-T1 mRNA and protein expression by NGN3+/CD133+ cells in exocrine pancreas tissue.

### NGN3 protein expression is positively regulated by TRKB signaling

**Fig. 3:**
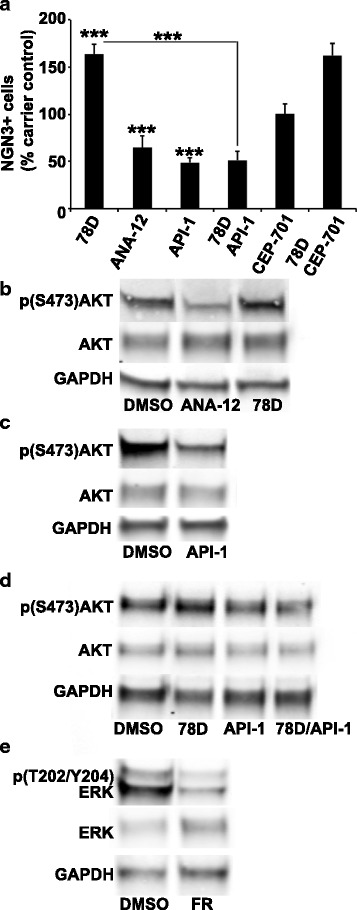
TRKB regulation of NGN3 in cultured human exocrine tissue. **a** Percentage of NGN3+ cells in human exocrine tissue cultures treated with Neurotrophic tyrosine kinase type 2 receptor (TRKB) agonist 7,8-dihydroxyflavone (78D, 0.1 μM), TRKB inhibitor ANA-12 (50 μM), protein kinase B (AKT) inhibitor API-1 (1 μM), combination of 78D and API-1, tyrosine kinase inhibitor CEP-701 (10 nM) or a combination of 78D and CEP-701 for 4 days. Mean ± SEM percentage of NGN3+ cells was determined using quantitative immunohistochemistry and normalized to dimethyl sulfoxide (DMSO) carrier control. Significance for ANA-12 determined by two-tailed homoscedastic Student’s *t*-test (*n* = 10 technical replica readings from 4 biological replicate exocrine cultures) and for other drugs by one-way ANOVA with Bonferroni post hoc testing (*n* = 10 technical replica readings from 3 biological replicate exocrine cultures), ***, *p* < 0.001. **b**-**d** Western blot analyses of signaling activity downstream of TRKB. **b** Levels of active protein kinase B (p(S473)AKT) following treatment with ANA-12 and 78D compared to DMSO carrier control. **c** Levels of active AKT following treatment with API-1 compared to DMSO carrier control. **d** Levels of active ERK1/2 (p(T202/Y204)ERK) following inhibition with FR180204 compared to DMSO carrier control. Protein loading level indicated by level of glyceraldehyde-3-phosphate dehydrogenase (GAPDH) in **b**-**e**, total AKT in **b**-**d** and total ERK in **e**

Drugs that selectively modulate receptor activity were used to determine if NGN3 is regulated by TRKB signaling. The percentage of NGN3+ cells significantly increased to a mean ± SEM of 170.5 ± 7.2 % of carrier control (*n* = 3, *p* = 2.6X10^-6^) following treatment of exocrine cultures with 7,8-dihydroxyflavone, an agonist that binds the TRKB extracellular domain (Fig. 3a). ANA-12, a TRKB ligand that inhibits activation and downstream signaling, significantly decreased the percentage of NGN3+ cells to 64.9 ± 5.2 % of carrier control (*n* = 4, *p* = 2.3X10^-5^). ANA-12 and 7,8-dihydroxyflavone had no significant effect on NGN3 mRNA expression (*n* = 3, *p* > 0.05). These significant and appropriate responses to TRKB-selective drugs of opposing action strongly suggest NGN3 protein is regulated by TRKB signaling.

### AKT is involved in the regulation of NGN3 by TRKB

Western blot analyses were used to determine if TRKB signaling involves changes in the phosphorylation state of AKT. Inhibition of TRKB with ANA-12 decreased AKT activation, as indicated by a decrease in phosphorylation of AKT serine 473 (p(S473)AKT), whereas 7,8-dihydroxyflavone increased p(S473)AKT (Fig. 3b). To determine if AKT is involved in the regulation of NGN3 by TRKB, exocrine cultures were treated with API-1, a pleckstrin domain-binding AKT inhibitor, alone and in combination with 7,8-dihydroxyflavone. Treatment with API-1 significantly decreased the mean ± SEM percentage of NGN3+ cells to 48.8 ± 9.7 % of carrier control (*n* = 3, *p* = 2.4X10^-4^) and decreased AKT activation (Fig. 3c). In combination with 7,8-dihydroxyflavone, API-1 blocked the increase in NGN3 observed with 7,8-dihydroxyflavone alone (*n* = 3, *p* = 4.8X10^-18^) (Fig. 3a). Similarly, API-1 blocked a significant (*n* = 3, *p* = 0.01) 7,8-dihydroxyflavone-dependent increase in AKT activation (representative blot shown in Fig. 3d). In addition to AKT, TRKB also can signal through ERK1/2 [36]. Treatment of exocrine tissue with ERK inhibitor FR180204 decreased ERK1/2 activation, as indicated by a decrease in phosphorylation of ERK1/2 threonine 202 and tyrosine 204 (p(T202/Y204)ERK) (Fig. 3e), but had no effect on the percentage of NGN3+ cells (*n* = 4, *p* > 0.05, Additional file 3: Figure S3). ANA-12, 7,8-dihydroxyflavone and API-1 did not have a significant effect on NGN3 mRNA expression (*n* = 4, *p* > 0.05, Additional file 4: Figure S4, Additional file 5: Figure S5 and Additional file 6: Figure S6).

To determine if TRKB-FL or other tyrosine kinases were involved in the regulation of NGN3, exocrine tissue was treated with tyrosine kinase inhibitor CEP-701. CEP-701 had no effect on NGN3 protein levels compared to carrier control (*n* = 3, *p* > 0.05) and in combination with 7,8-dihydroxyflavone, did not block the increase in NGN3 observed following treatment with 7,8-dihydroxyflavone alone (*n* = 3, *p* > 0.05) (Fig. 3a). CEP-701 also had no significant effect on NGN3 mRNA expression (*n* = 3, *p* > 0.05, Additional file 7 Figure S7). These results demonstrate that AKT is involved in the regulation of NGN3 by TRKB and, in agreement with low levels of TRKB-FL expression, suggest that TRKB tyrosine kinase activity is not required.

TRKB signaling can lead to proliferation and invasiveness of pancreatic adenocarcinoma cell lines [13–15]. While mRNA expression of cellular proliferation antigen Ki-67 and incorporation of 5-ethynyl-2’-deoxyuridine (EdU) both increased during proliferation of isolated NGN3+ cells, there was little evidence of cell proliferation as measured by either technique in cultures of intact exocrine tissue [5] or following pharmacological modulation of TRKB or AKT activity (mean C_t_ >35, *n* = 4). This data, along with coexpression of with NGN3 in histologically normal pancreas biopsies, suggest TRKB signaling results in *de novo* NGN3 protein accumulation rather than NGN3+ cell proliferation.

NGN3 transcription and protein half-life are regulated by Notch signaling through the transcriptional regulation of repressor hairy and enhancer of split-1 (HES1) [1, 4, 37–39]. Neither TRKB agonist 7,8-dihydroxyflavone nor antagonist ANA-12 had a significant effect on HES1 mRNA levels (*p* > 0.05, *n* = 4, Additional file 4: Figures S4, Additional file 5: Figure S5), which suggests regulation of NGN3 by TRKB is independent of canonical HES1-mediated Notch signaling.

### Differential expression of genes involved in neurotrophin signaling

The expression level of 320 genes involved in, or regulated by, neurotrophin signaling were analyzed in NGN3+/CD133+ and NGN3/CD133D cell transcriptomes (genes listed in Additional file 8 Table S8). The percentage of genes differentially expressed by more than 2-fold in the 320 gene set was significantly higher than an equal sized collection of genes randomly selected from the expression dataset (*n* = 10, *p* = 0.0001). In addition to TRKB itself, this analysis identified 41 genes upregulated and 54 genes down regulated by more than 2-fold in the NGN3+/CD133+ population (Table 1). A 187-fold increase in expression of guanine nucleotide exchange factor 3 (VAV3) suggests involvement of Rho/Rac GTPases, as VAV3 can function as an intermediate between TRKB signaling and GTPase activation [40–43]. Neural cell adhesion molecule 1 (NCAM1), which is upregulated 184-fold, also is involved in modulation of TRKB signaling [44, 45]. Significant upregulation of these genes suggest possible downstream targets of TRKB signaling in NGN3+ exocrine cells.

**Table 1:** Transcriptome analysis of neurotrophin signaling in cultured human exocrine tissue

Upregulated		Down regulated			
Symbol	Fold	Symbol	Fold	Symbol	Fold
**NTRK2**	**277.1**	IL6	36.4	EIF4EBP1	2.3
**VAV3**	**187.6**	KCNN2	28.8	MYC	2.3
**NCAM1**	**184.0**	MEF2C	19.2	SHC1	2.3
BCL2	42.1	NRG4	18.4	RGS19	2.2
CCND1	22.5	GRIA3	17.9	IL6R	2.2
PLCG2	20.7	SH3GL2	16.8	MAP2K7	2.2
SPP1	19.7	MAP3K5	16.7	GNB2L1	2.1
NPY1R	18.8	GFRA1	15.7	CDK5R1	2.1
RPS6KA5	10.4	GDNF	15.4	PIK3R3	2.1
CNR1	9.4	KSR1	14.7	HSPB1	2.0
TGFA	7.1	GRPR	14.0	RPS6	2.0
NCF2	5.5	TGFB1	12.6	EGR2	2.0
IRAK2	5.5	GMFG	11.7		
TRO	5.2	UCN	11.6		
IRS2	5.1	IRAK3	11.1		
DOK5	4.9	RAB3A	8.0		
MAPK10	4.3	NPFFR2	6.8		
NCK2	4.2	PIK3CG	5.8		
FYN	3.7	ARHGDIB	5.0		
DNM2	3.3	CBLN1	5.0		
RIT1	3.0	IL10RA	4.9		
NFKBIE	2.9	SH2B3	4.4		
MT3	2.9	LIFR	4.3		
SHC4	2.7	NELL1	3.8		
DPYSL2	2.7	EEF2	3.7		
GABRB3	2.6	CX3CR1	3.6		
FGF9	2.5	LINGO1	3.2		
DOCK3	2.3	PTGER2	3.2		
ITPR3	2.3	RPS6KA6	3.1		
PIK3R1	2.2	FGFR1	3.1		
SIRPA	2.2	STAT5A	2.9		
GAB1	2.2	KRAS	2.8		
PIK3CD	2.2	CRTC1	2.8		
CALM2	2.1	MAPT	2.7		
HRAS	2.1	FGF2	2.6		
NGFRAP1	2.1	ATF4	2.6		
NCK1	2.1	ELMO1	2.5		
PRKCZ	2.1	AP2A1	2.5		
DYNLT1	2.1	MAPK11	2.5		
DNAL4	2.1	JAK2	2.4		
MAP3K1	2.0	STAT5B	2.4		
AKT3	2.0	MAP2K2	2.3		

Transcriptome data from paired NGN3+/CD133+ and NGN3/CD133-depleted cell populations isolated from 3 biological replicate exocrine cultures. Data was analyzed for expression of 320 genes annotated as being involved with, or downstream of, neurotrophin signaling (full gene list in Additional file 8: Table S8). Gene symbol, name and fold expression in NGN3+/CD133+ cells are shown ranked by fold expression compared to NGN3/CD133-depleted cells. Genes discussed shown in bold. Only genes with a ≥2-fold difference are shown

## Discussion

TRKB-FL activation results in intrinsic tyrosine kinase activity, adapter protein docking and downstream signal transduction. Low levels of TRKB-FL expression and failure of tyrosine kinase inhibition to block regulation of NGN3 suggest involvement of isoforms TRKB-T1, TRKB-T-Shc or TRKB-N-T1. TRKB-T1 is the predominant isoform expressed in the adult rodent central nervous system [46] and is expressed in pancreatic cancer cell lines, but was not detected in normal human pancreatic tissue or immortalized ductal epithelial cells [12]. However, transcriptome-based gene splicing analysis, isoform-specific quantitative RTPCR, western blot and immunohistochemistry demonstrate TRKB-T1 expression by NGN3+ cells in cultured human exocrine tissue and histologically normal cadaveric pancreas biopsies. Although there is evidence for transcription of TRKB-T-Shc and TRKB-N-T1 in the NGN3+/CD133+ cell transcriptome, TRKB-T-Shc lacks an activation domain and functions as a negative regulator of TRKB-FL rather than involving AKT signaling [23]. Cytoplasmic localization of TRKB-N-T1 [23] and absence of domains required for TRKB agonist interaction [47] conflict with observed cell-surface antibody staining and signaling results, and argue against involvement of TRKB-N-T1. Taken together, the expression and signaling results reported here are most consistent with regulation of NGN3 by TRKB-T1. The failure of drug treatments to significantly affect NGN3 mRNA levels suggests that TRKB signaling regulates NGN3 translational efficiency or protein stability.

While NGN3 plays a critical role in endocrine lineage fate commitment during pancreas development, its role in the adult pancreas is largely unknown. Although it cannot be detected in the rodent pancreas, targeted disruption of murine NGN3 has a negative impact on islet function [48]. Upregulation of NGN3 by pancreatic endocrine and exocrine cells dedifferentiated under physiological stress conditions suggest it plays a role in cellular plasticity [49–51]. Given the role of TRKB in neuronal plasticity [52, 53], it may similarly participate in the initiation or maintenance of exocrine cell dedifferentiation through positive regulation of NGN3.

Although beta cells within preexisting islets [54–60] have been shown to be the predominant source of regenerating beta cells under normal circumstances and following certain types of pancreatic injury [54–59], it is becoming increasingly evident that pancreatic exocrine cells can be reprogrammed to an endocrine cell fate. Exocrine cells have the capacity to generate insulin-expressing cells following injury and in vitro manipulation [61–70]. Transient expression of NGN3 through adenoviral transduction [71–73], partial duct ligation [74, 75], 90 % pancreatectomy [76, 77], in vivo delivery of cytokines [78, 79] and stabilization of NGN3 protein through knockout of GSK3β [76] or the E3 ubiquitin ligase FBXW7 [80] demonstrate that exocrine cells in the adult pancreas have the capacity to take on an endocrine cell fate and strongly suggest a role for NGN3 in this process. In this regard, pharmacological control of NGN3 through TRKB signaling may represent an innovative approach to the treatment of diabetes.

## Conclusion

Approximately 30 % of total and 95 % of NGN3+ cells in primary cultures of adult human exocrine pancreas tissue expresses TRKB. TRKB-T1, which lacks a tyrosine kinase domain, is the predominant isoform expressed in cultured human exocrine tissue and is expressed in histologically normal cadaveric pancreas biopsies. TRKB signaling positively regulates NGN3 independent of tyrosine kinase activity in cultured adult human exocrine tissue without significantly affecting NGN3 mRNA levels. Identification of VAV3 overexpression in the NGN3+ cell transcriptome suggests TRKB signaling may also involve Rho/Rac GTPases.

## Additional files

Additional file 1: Figure S1. TRKB/NGN3 FACS gating strategy and controls. All FACS analyses used to determine the coexpression of TRKB and NGN3 presented in Fig. 1a–d. (PDF 2250 kb) Additional file 2: Figure S2. TRKB/CD133 FACS gating strategy and controls. All FACS analyses used to determine the coexpression of TRKB and CD133 presented in Fig. 1e–h. (PDF 2833 kb) Additional file 3: Figure S3. Level of NGN3 protein following inhibition of ERK1/2. Results of quantitative immunohistochemistry following treatment of four biological replicate samples (A–D) of exocrine tissue with DMSO or ERK1/2 inhibitor FR180204 for four days. Mean and standard error of the mean (SEM) shown as a percentage of total nuclei (%NGN3) and as percentage of DMSO control (% control). Mean and SEM of % control and results of Student’s *t*-test (TTEST) performed on %NGN3 indicated at right. (DOCX 48 kb) Additional file 4: Figure S4. Expression of NGN3 and hairy and enhancer of split-1 (HES1) mRNA following treatment with TRKB antagonist ANA-12. Quantitative RTPCR results of four biological replicate samples (A–D) treated with ANA-12 for four days. RQ, relative quantification of NGN3 and HES1 normalized to the level of cyclophillin A. ΔCt, change in threshold cycle (Ct). Mean and standard error of the mean (SEM), results of Student’s *t*-test (TTEST) and percentage of control are shown. (DOCX 50 kb) Additional file 5: Figure S5. Expression of NGN3 and hairy and enhancer of split-1 (HES1) mRNA following treatment with TRKB agonist 7,8 dihydroxyflavone (78D). Quantitative RTPCR results of four biological replicate samples (A–D) treated with 78D for four days. RQ, relative quantification of NGN3 and HES1 normalized to the level of cyclophillin A. ΔCt, change in threshold cycle (Ct). Mean and standard error of the mean (SEM), results of Student’s *t*-test (TTEST) and percentage of control are shown. (DOCX 50 kb) Additional file 6: Figure S6. Expression of NGN3 and hairy and enhancer of split-1 (HES1) mRNA following treatment with AKT inhibitor API-1. Quantitative RTPCR results of four biological replicate samples (A–D) treated with API-1 for four days. RQ, relative quantification of NGN3 and HES1 normalized to the level of cyclophillin A. ΔCt, change in threshold cycle (Ct). Mean and standard error of the mean (SEM), results of Student’s *t*-test (TTEST) and percentage of control are shown. (DOCX 49 kb) Additional file 7: Figure S7. Expression of NGN3 and hairy and enhancer of split-1 (HES1) mRNA following treatment with tyrosine kinase inhibitor CEP-701. Quantitative RTPCR results of four biological replicate samples (A–D) treated with API-1 for four days. RQ, relative quantification of NGN3 and HES1 normalized to the level of cyclophillin A. ΔCt, change in threshold cycle (Ct). Mean and standard error of the mean (SEM), results of Student’s *t*-test (TTEST) and percentage of control are shown. (DOCX 47 kb) Additional file 8: Table S8. Genes involved with neurotrophin signaling. List of 320 genes annotated as being involved with, or downstream of, neurotrophin signaling. (DOCX 148 kb)

## Abbreviations

AKT: Protein kinase B
ANA-12: N-[2-[[(Hexahydro-2-oxo-1H-azepin-3-yl)amino]carbonyl]phenyl]benzo[b]thiophene-2-carboxamide
ANOVA: Analysis of variance
API-1: 4-Amino-5,8-dihydro-5-oxo-8-β-D-ribofuranosyl-pyrido[2,3-d]pyrimidine-6-carboxamide
BSA: Bovine serum albumin
CEP-701: (9S, 10S,12R)-2,3,9,10,11,12-Hexahydro-10-hydroxy-10-(hydroxymethyl)-9-methyl-9,12-epoxy-1H-diindolo[1,2,3-fg:3’,2’,1’-kl]pyrrolo[3,4-i][1, 6]benzodiazocin-1-one
DMSO: Dimethyl sulfoxide
EDTA: Ethylenediaminetetraacetic acid
EdU: 5-ethynyl-2’-deoxyuridine
ERK: Extracellular signal related kinase
FACS: Fluorescence activated cell sorting. C_t_, threshold cycle
FR180204: 5-(2-Phenyl-pyrazolo[1,5-a]pyridin-3-yl)-1H-pyrazolo[3,4-c]pyridazin-3-ylamine
HES1: Repressor hairy and enhancer of split-1
kDa: Kilodalton
NCAM1: Neural cell adhesion molecule 1
NGN3: Neurogenin 3
PBS: Phosphate buffered saline
PE: Phycoerythrin
PPIA: cyclophillin A
RTPCR: Reverse transcriptase polymerase chain reaction
TRKB: Neurotrophic tyrosine kinase receptor type 2
VAV3: Guanine nucleotide exchange factor 3
