# Supplementary figures and images for: Neurogenin 3 is regulated by neurotrophic tyrosine kinase receptor type 2 (TRKB) signaling in the adult human exocrine pancreas
Source: Cell Commun Signal. 2016 Sep 22;14:23. doi: 10.1186/s12964-016-0146-x (PMC5034529; doi:10.1186/s12964-016-0146-x)

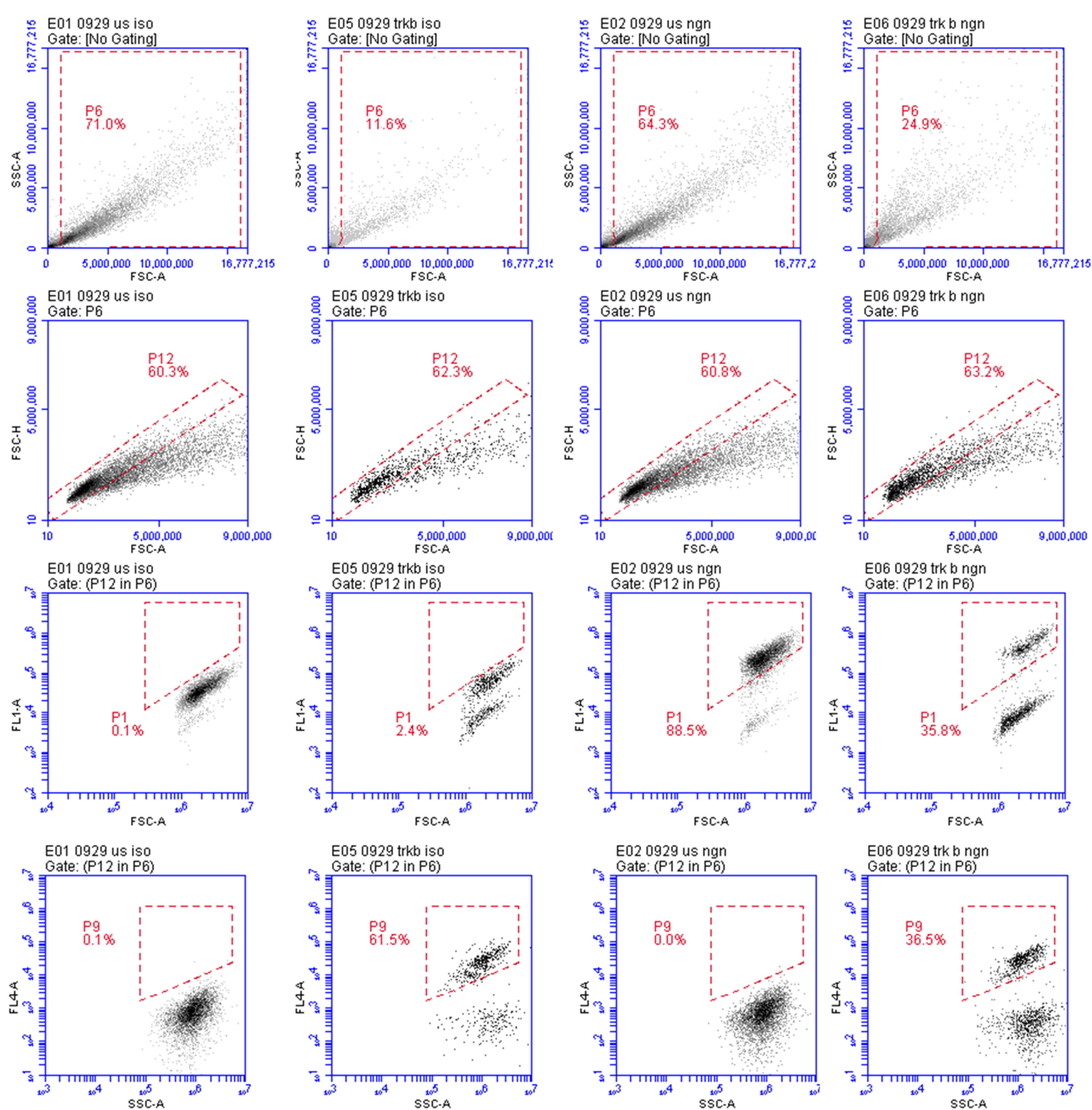

Supplement: Additional file 1: Figure S1. — TRKB/NGN3 FACS gating strategy and controls. All FACS analyses used to determine the coexpression of TRKB and NGN3 presented in Fig. 1a–d. (PDF 2250 kb) [file 12964_2016_146_MOESM1_ESM.pdf]

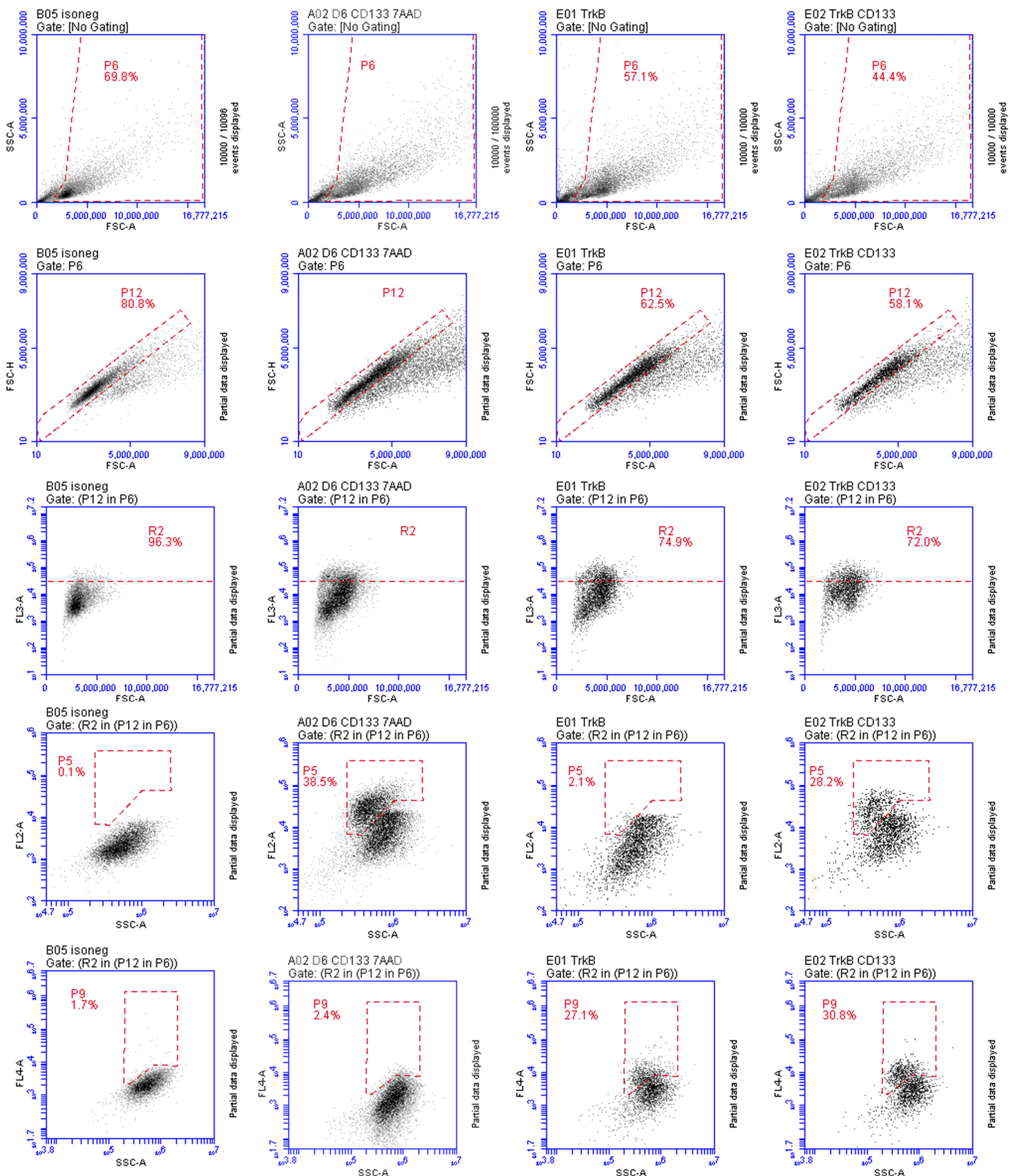

Supplement: Additional file 2: Figure S2. — TRKB/CD133 FACS gating strategy and controls. All FACS analyses used to determine the coexpression of TRKB and CD133 presented in Fig. 1e–h. (PDF 2833 kb) [file 12964_2016_146_MOESM2_ESM.pdf]
