# Supplementary material for: Neurogenin 3 is regulated by neurotrophic tyrosine kinase receptor type 2 (TRKB) signaling in the adult human exocrine pancreas
Source: Cell Commun Signal. 2016 Sep 22;14:23. doi: 10.1186/s12964-016-0146-x (PMC5034529; doi:10.1186/s12964-016-0146-x)
Supplement: Additional file 3: Figure S3. — Level of NGN3 protein following inhibition of ERK1/2. Results of quantitative immunohistochemistry following treatment of four biological replicate samples (A–D) of exocrine tissue with DMSO or ERK1/2 inhibitor FR180204 for four days. Mean and standard error of the mean (SEM) shown as a percentage of total nuclei (%NGN3) and as percentage of DMSO control (% control). Mean and SEM of % control and results of Student’s t-test (TTEST) performed on %NGN3 indicated at right. (DOCX 48 kb) [file 12964_2016_146_MOESM3_ESM.docx]

|  |  | %NGN3 |  |  | as % control | |  |  |
| --- | --- | --- | --- | --- | --- | --- | --- | --- |
| Sample ID |  | Mean | SEM |  | %NGN3 | SEM |  |  |
| A | DMSO | 15.16 | 1.23 |  | 100.00 | 17.37 |  |  |
| B | DMSO | 19.98 | 0.87 |  | 100.00 | 4.36 | MEAN | SEM |
| C | DMSO | 23.96 | 3.15 |  | 100.00 | 13.14 | 105.2 | 6.3 |
| D | DMSO | 27.45 | 2.32 |  | 100.00 | 8.46 |  |  |
| A | FR180204 | 14.19 | 1.50 |  | 93.58 | 9.91 | TTEST | 0.68 |
| B | FR180204 | 23.96 | 2.52 |  | 106.83 | 11.25 |  |  |
| C | FR180204 | 27.61 | 3.51 |  | 115.25 | 14.66 |  |  |
| D | FR180204 | 27.88 | 2.41 |  | 101.55 | 8.79 |  |  |
