# Supplementary material for: Neurogenin 3 is regulated by neurotrophic tyrosine kinase receptor type 2 (TRKB) signaling in the adult human exocrine pancreas
Source: Cell Commun Signal. 2016 Sep 22;14:23. doi: 10.1186/s12964-016-0146-x (PMC5034529; doi:10.1186/s12964-016-0146-x)
Supplement: Additional file 4: Figure S4. — Expression of NGN3 and hairy and enhancer of split-1 (HES1) mRNA following treatment with TRKB antagonist ANA-12. Quantitative RTPCR results of four biological replicate samples (A–D) treated with ANA-12 for four days. RQ, relative quantification of NGN3 and HES1 normalized to the level of cyclophillin A. ΔCt, change in threshold cycle (Ct). Mean and standard error of the mean (SEM), results of Student’s t-test (TTEST) and percentage of control are shown. (DOCX 50 kb) [file 12964_2016_146_MOESM4_ESM.docx]

|  |  | RQ | | ΔCt | |  |  |
| --- | --- | --- | --- | --- | --- | --- | --- |
|  | Sample ID | NGN3 | HES1 | NGN3 | HES1 |  |  |
| DMSO | A | 1.00 | 1.00 | 4.79 | 1.27 |  |  |
|  | B | 1.00 | 1.00 | 8.35 | 2.23 |  |  |
|  | C | 1.00 | 1.00 | 5.50 | 0.14 |  |  |
|  | D | 1.00 | 1.00 | 7.44 | 0.63 |  |  |
| ANA-12 | A | 0.17 | 1.14 | 7.37 | 1.08 |  |  |
|  | B | 0.61 | 0.65 | 9.07 | 2.85 |  |  |
|  | C | 0.32 | 0.55 | 7.13 | 1.01 |  |  |
|  | D | 0.68 | 1.08 | 8.00 | 0.52 |  |  |
|  |  |  |  |  |  |  |  |
|  |  |  |  |  |  |  |  |
|  | Mean DMSO |  |  | 6.52 | 1.07 |  |  |
|  | SE DMSO |  |  | 0.83 | 0.45 |  |  |
|  | Mean ANA12 | 0.44 | 0.85 | 7.89 | 1.37 |  |  |
|  | SE ANA12 | 0.12 | 0.15 | 0.44 | 0.51 |  |  |
|  | TTEST |  |  | 0.19 | 0.68 |  |  |
|  |  |  |  |  |  |  |  |
