# Supplementary material for: Neurogenin 3 is regulated by neurotrophic tyrosine kinase receptor type 2 (TRKB) signaling in the adult human exocrine pancreas
Source: Cell Commun Signal. 2016 Sep 22;14:23. doi: 10.1186/s12964-016-0146-x (PMC5034529; doi:10.1186/s12964-016-0146-x)
Supplement: Additional file 6: Figure S6. — Expression of NGN3 and hairy and enhancer of split-1 (HES1) mRNA following treatment with AKT inhibitor API-1. Quantitative RTPCR results of four biological replicate samples (A–D) treated with API-1 for four days. RQ, relative quantification of NGN3 and HES1 normalized to the level of cyclophillin A. ΔCt, change in threshold cycle (Ct). Mean and standard error of the mean (SEM), results of Student’s t-test (TTEST) and percentage of control are shown. (DOCX 49 kb) [file 12964_2016_146_MOESM6_ESM.docx]

|  |  | RQ | | ΔCt | |  |  |
| --- | --- | --- | --- | --- | --- | --- | --- |
|  | Sample ID | NGN3 | HES1 | NGN3 | HES1 |  |  |
| DMSO | A | 1.00 | 1.00 | 6.53 | 1.54 |  |  |
|  | B | 1.00 | 1.00 | 8.35 | 2.23 |  |  |
|  | C | 1.00 | 1.00 | 5.50 | 0.14 |  |  |
|  | D | 1.00 | 1.00 | 7.44 | 0.63 |  |  |
| API-1 | A | 2.50 | 1.46 | 5.20 | 0.99 |  |  |
|  | B | 4.37 | 1.77 | 6.23 | 1.41 |  |  |
|  | C | 0.33 | 1.63 | 7.11 | -0.56 |  |  |
|  | D | 1.73 | 0.54 | 6.65 | 1.51 |  |  |
|  |  |  |  |  |  |  |  |
|  |  |  |  |  |  |  |  |
|  | Mean DMSO |  |  | 6.96 | 1.13 |  |  |
|  | SEM DMSO |  |  | 0.61 | 0.47 |  |  |
|  | Mean API-1 | 2.23 | 1.35 | 6.30 | 0.84 |  |  |
|  | SEM API-1 | 0.84 | 0.28 | 0.41 | 0.48 |  |  |
|  | TTEST |  |  | 0.40 | 0.67 |  |  |
|  |  |  |  |  |  |  |  |
