# Supplementary material for: Neurogenin 3 is regulated by neurotrophic tyrosine kinase receptor type 2 (TRKB) signaling in the adult human exocrine pancreas
Source: Cell Commun Signal. 2016 Sep 22;14:23. doi: 10.1186/s12964-016-0146-x (PMC5034529; doi:10.1186/s12964-016-0146-x)
Supplement: Additional file 8: Table S8. — Genes involved with neurotrophin signaling. List of 320 genes annotated as being involved with, or downstream of, neurotrophin signaling. (DOCX 148 kb) [file 12964_2016_146_MOESM8_ESM.docx]

**Neurogenin 3 is regulated by TrkB in the human pancreas**

Michael J. Shamblott, Marci O’Driscoll, Danielle L. Gomez, Dustin McGuire

Table S3

| **Symbol** | | | | | | | |
| --- | --- | --- | --- | --- | --- | --- | --- |
| ABL1 | CCND1 | EGR1 | GRPR | MAP3K3 | NPFFR2 | PTK2B | SOS1 |
| ACACB | CD40 | EGR2 | GSK3B | MAP3K5 | NPY | PTPN11 | SOS2 |
| ADAM17 | CDC42 | EHD4 | HCRT | MAPK1 | NPY1R | PTPRF | SPP1 |
| ADCYAP1R1 | CDH2 | EIF2S1 | HRAS | MAPK10 | NPY2R | RAB3A | SQSTM1 |
| AKT1 | CDK5 | EIF2S2 | HSPB1 | MAPK11 | NRAS | RAC1 | SRC |
| AKT2 | CDK5R1 | EIF4E | IGF2BP1 | MAPK12 | NR1I2 | RAF1 | STAT1 |
| AKT3 | CDKL5 | EIF4EBP1 | IKBKB | MAPK13 | NRG1 | RANBP9 | STAT2 |
| ALPL | CFL1 | ELK1 | IL10 | MAPK14 | NRG2 | RAP1A | STAT3 |
| AP2A1 | CLTA | ELMO1 | IL10RA | MAPK3 | NRG4 | RAP1B | STAT4 |
| AP2A2 | CLTC | FAIM | IL1B | MAPK7 | NSF | RAPGEF1 | STAT5A |
| AP2B1 | CNR1 | FAS | IL1R1 | MAPK8 | NTF3 | RASA1 | STAT5B |
| AP2M1 | CNTF | FASLG | IL6 | MAPK9 | NTF4 | RASGRF1 | SYN1 |
| AP2S1 | CNTFR | FGF2 | IL6R | MAPKAPK2 | NTRK1 | RELA | TACR1 |
| APC | CREB1 | FGF9 | IL6ST | MAPT | NTRK2 | RGS19 | TFG |
| ARHGDIA | CREB3 | FGFR1 | IRAK1 | MARCKS | NTRK3 | RHOA | TGFA |
| ARHGDIB | CREB3L4 | FOS | IRAK2 | MATK | NTSR1 | RHOG | TGFB1 |
| ARTN | CRH | FOXO3 | IRAK3 | MC2R | PAK1 | RIPK2 | TIAM1 |
| ATF4 | CRHBP | FRS2 | IRAK4 | MCF2L | PDK1 | RIT1 | TP53 |
| BAD | CRHR1 | FRS3 | IRS1 | MEF2A | PDPK1 | RIT2 | TP73 |
| BAX | CRHR2 | FUS | IRS2 | MEF2C | PIK3CA | RPS6 | TRAF6 |
| BCL2 | CRK | FYN | IRS4 | MIR3661 | PIK3CB | RPS6KA1 | TRO |
| BCL2L1 | CRKL | GAB1 | ITPR1 | MRAS | PIK3CD | RPS6KA2 | TSC2 |
| BCL2L11 | CRTC1 | GAB2 | ITPR2 | MT3 | PIK3CG | RPS6KA3 | UCN |
| BDNF | CSDE1 | GABRB3 | ITPR3 | MTOR | PIK3R1 | RPS6KA4 | VAV2 |
| BMP2 | CSK | GALR1 | JAK2 | MYC | PIK3R2 | RPS6KA5 | VAV3 |
| BRAF | CSNK2A1 | GALR2 | JUN | NCAM1 | PIK3R3 | RPS6KA6 | VGF |
| CALM1 | CTNNB1 | GDNF | KCNA3 | NCF2 | PIK3R4 | RPS6KB1 | YBX1 |
| CALM2 | CX3CR1 | GFRA1 | KCNN2 | NCK1 | PIK3R5 | RRAS | YWHAB |
| CALM3 | CXCR4 | GFRA2 | KIDINS220 | NCK2 | PLCG1 | RRAS2 | YWHAE |
| CALML3 | CYFIP1 | GFRA3 | KRAS | NEDD4L | PLCG2 | SH2B1 | YWHAG |
| CALML5 | DLG1 | GIPC1 | KSR1 | NELL1 | PNOC | SH2B2 | YWHAH |
| CALML6 | DNAJA3 | GMFB | LIF | NF1 | PPP2CA | SH2B3 | YWHAQ |
| CAMK1 | DNAL4 | GMFG | LIFR | NFATC4 | PRDM4 | SH3GL2 | YWHAZ |
| CAMK2A | DNM1 | GNB2L1 | LINGO1 | NFKB1 | PRKAA1 | SHC1 | ZNF274 |
| CAMK2B | DNM2 | GRB2 | MAGED1 | NFKBIA | PRKAA2 | SHC2 | ZFP91 |
| CAMK2D | DOCK1 | GRIA1 | MAP2K1 | NFKBIB | PRKCD | SHC3 |  |
| CAMK2G | DOCK3 | GRIA2 | MAP2K2 | NFKBIE | PRKCI | SHC4 |  |
| CAMK4 | DOK5 | GRIA3 | MAP2K5 | NGF | PRKCZ | SIRPA |  |
| CASP3 | DPYSL2 | GRIN1 | MAP2K7 | NGFR | PSEN1 | SNORD95 |  |
| CBLN1 | DYNLT1 | GRIN2B | MAP3K1 | NGFRAP1 | PSPN | SNORD96A |  |
| CCKAR | EEF2 | GRIP1 | MAP3K2 | NPFF | PTGER2 | SORT1 |  |
